# Comparisons of microvascular and macrovascular changes in aldosteronism-related hypertension and essential hypertension

**DOI:** 10.1038/s41598-017-02622-2

**Published:** 2017-06-01

**Authors:** Monica Varano, Pierluigi Iacono, Massimiliano M. Tedeschi, Claudio Letizia, Mario Curione, Claudio Savoriti, Erika Baiocco, Laura Zinnamosca, Cristiano Marinelli, Barbara Boccassini, Mariacristina Parravano

**Affiliations:** 1 G. B. Bietti Foundation, IRCCS, Rome, Italy; 2 Department of Internal Medicine and Medical Specialties, Unit of Secondary Arterial Hypertension, University “La Sapienza”, Rome, Italy; 3 Department of Internal Medicine and Medical Specialties, University “La Sapienza”, Rome, Italy

## Abstract

Case-control observational study to evaluate the microvascular and macrovascular changes in patients with hypertension secondary to primary aldosteronism (PA), essential hypertension (EH) and healthy subjects. Measurements of arterial stiffness including augmentation index (AIx) and pulse wave velocity (PWV) were assessed using a TensioClinic arteriograph system. Retinal microcirculation was imaged by a Retinal Vessel Analyzer (RVA) and a non-midriatic camera (Topcon-TRC-NV2000). IMEDOS software analyzed the retinal artery diameter (RAD), retinal vein diameters (RVD) and arteriole-to-venule ratio (AVR) of the vessels coming off the optic disc. Thirty, 39 and 35 patients were included in the PA, EH and control group, respectively. The PA group showed higher PWV values compared only with the control group. The mean brachial and aortic AIx values did not show significant difference between groups. In the PA group, the mean RVD and AVR values were significantly lower than in the EH and control groups, whereas the parameters did not differ between the EH and control groups. In conclusion, AVR appears significantly modified in the PA group compared with the EH group and could represent an early and more reliable indicator of microvascular remodeling.

## Introduction

Primary aldosteronism (PA) is a common cause of arterial hypertension and represents the most frequent form of secondary hypertension^1^. PA has been associated with a higher incidence of cardiovascular events than essential hypertension (EH), due to aldosterone pressure-independent effects, with marked target organ damage affecting the heart, carotid artery and kidneys^2^.

Recent studies of patients affected by PA have demonstrated an increased accumulation of collagen, especially type III, even in small arteries and arterioles, compared with normotensive and essential hypertensive subjects^3, 4^. These changes in extracellular matrix are able to modify profoundly the microvascular structure and play an important role in the development of cardiovascular fibrosis and the consequent increase in the rigidity of such structures. The etiopathogenetic mechanism by which aldosterone is able to effect such changes has not yet been completely identified, although it is known that aldosterone helps substantially in the accumulation of different collagen fibers and growth factors in vascular walls in patients with PA^4, 5^.

To date, the only possible *in vivo* evaluation of these changes involves an invasive approach through biopsy^4^. For this reason, there is currently considerable interest in research on the development of non-invasive methods used in the study of vascular remodeling and arterial stiffness alterations. Arterial stiffness seems to be a predictor of future cardiovascular events in hypertensive patients and is especially high in PA compared with EH^6–9^.

The retina represents a unique site where the microvasculature can be directly and non-invasively visualized. Retinal microvasculature assessment offers an opportunity for detailed *in vivo* study of the structure of small arterioles and venules and of their modifications in relation to systemic disease processes such as hypertension and diabetes.

Despite the increasing use of retinal vascular imaging in evaluating the alterations of retinal microvascularization in patients with EH and the demonstrated homology between retinal microvascular morphology and peripheral small resistant arteries^10^, no information is available regarding the retinal microvascular changes associated with PA and their correlation with the macrovascular compartment.

On the basis of the different pathogenetic mechanisms of arterial hypertension secondary to PA and in the EH, the current pilot study aimed to provide a preliminary analysis of the modifications in microvascular retinal circulation and in the macrovascular compartment in patients with arterial hypertension related to PA and EH.

## Results

Demographic characteristics of patients with PA, EH and the subjects in the control group are shown in Table 1. Thirty and 39 patients were included in the PA and EH groups, respectively. Thirty-five age-matched healthy volunteers (HS) of both sexes, without known cardiovascular, renal, or heapatic disorders, were selected as controls.

**Table 1:** Retinal microvascular and systemic macrovascular assessment in hypertension secondary to primary aldosteronism.

	Essential Hypertension (EH) (N 39)	Primary Aldosteronism (PA) (N 30)	Control group (N 35)	F ratio (p value, ANOVA statistic)/Chi-square test (p:value)
Age (years)	55.8 ± 9.5	54.6 ± 11,6	53.1 ± 6,9	0,816 (p:0.44)
Gender (F/M)	23/16	17/13	21/14	0.07 (p:0.96)
Body Mass Index (kg.m^−2^)	25.6 ± 3.4 (p: <0.05 vs control) (p:ns vs PA)	26.2 ± 4.32 (p: <0.05 vs control)	23.4 ± 2.5	5.86 (p:0.004)
Mean time of known duration of hypertension (years)	2.8 ± 2.2 (p: 0.56 vs PA, t-test)	3.1 ± 2.1		
Smokers	8/39 (20%)	4/30 (13.3%)	5/35 (14%)	0.803 (p:0.66)
Medications				
**Anti-ipertensive**				8.41 (p:0.13)
ACE-I use,	8	5		
ARB use,	12	9		
Anti-ALDO	1	9		
CCB use,	13	16		
Diuretic use	16	11		
β-Blocker use	7	7		
**Statins**	2	2		
SBP clin (mmHg)*	132.2 ± 13.1 (p: <0.05 vs control) (p:ns vs PA)	132.2 ± 13 (p: <0.05 vs control)	120.6 ± 10	10.24 (p:<0.001)
DBP clin (mmHg)^†^	81.5 ± 8.7 (p: <0.05 vs control) (p:ns vs PA)	83 ± 8.4 (p: <0.05 vs control)	75.4 ± 8.4	7.42 (p:0.001)
ABPM-SBP (mmHg)^‡^	129.5 ± 11.1 (p: <0.05 vs control) (p:ns vs PA)	134.4 ± 17 (p: <0.05 vs control)	117.5 ± 9.6	19.55 (p:<0.001)
ABPM-DBP (mmHg)	79.1 ± 6.3 (p: <0.05 vs control) (p:ns vs PA)	81.7 ± 10.8 (p: <0.05 vs control)	71.7 ± 9.4	15.74 (p:<0.001)
ABPM-HR (bpm)^§^	68.8 ± 9.0	70.3 ± 10.1	68,7 ± 8,1	0.29 (p:0.74)
ABPM-MAP (mean arterial pressure)^||^ (mmHg)	97.3 ± 8.2 (p: <0.05 vs control) (p:ns vs PA)	99.3 ± 12.5 (p: <0.05 vs control)	89.7 ± 9.3	8.51 (p:<0.001)
ABPM-PP (pulse pressure) (mmHg)^#^	49.9 ± 8.3 (p: <0.05 vs control) (p:ns vs PA)	52.7 ± 9.1 (p: <0.05 vs control)	45.5 ± 7.8	6.50 (p:0.002)
ABP (mmHg)	125.4 ± 11.8 (p: <0.05 vs control) (p:ns vs PA)	128.9 ± 17.4 (p: <0.05 vs control)	116 ± 12	6.98 (p:0.001)
Glycemia (mg/dL)	94 ± 18 (p:ns vs PA) (p: ns vs control)	92 ± 13 (p: ns vs control)	90 ± 10	0.33 (p:0.70)
Serum potassium (mEq/dL)	4.27 ± 0.37 (p:ns vs PA) (p: ns vs control)	3.99 ± 0.68 (p: ns vs control)	4.21 ± 0.42	0.97 (p:0.43)
Serum Creatinine (mg/dL)	0.86 ± 0.15 (p:0.05 vs PA) (p: ns vs control)	1 ± 0.19 (p: <0.05 vs control)	0.88 ± 0.16	2.34 (p:0.03)
Plasma aldosterone (PAC) (ng/dl)	104 ± 60 (p: <0.05 vs PA) (p: 0.ns vs control)	426 ± 422 (p: <0.05 vs control)	85 ± 32	8.77 (p:0.001)
PAC/PRA (plasma renin activity) ratio (ng/dl:ng/ml/h)	17.6 ± 15 (p: <0.05 vs PA) (p: 0.ns vs control)	84.3 ± 79 (p: <0.05 vs control)	6.6 ± 2.1	10.21 (p:0.001)

Demographic characteristics, clinical data and laboratory testing of the study groups. *Systolic Blood Pressure Clinostatism (SBP clin), ^†^Diastolic Blood Pressure Clinostatism (DBP clin), ^‡^ambulatory blood pressure monitoring (ABPM), ^§^Heart Rate (HR), ^||^Mean Arterial Pressure (MAP), ^#^Pulse Pressure (PP), Aortic Blood Pressure (ABP). Medications: ARB: angiotensin receptor blocker. ACE-I: ACE inhibitor; CCB: calcium channel blocker, ANTI-ALDO: anti aldosteronic drugs.

There were no significant differences in age, gender and smoking habits between the groups; the body mass index was significantly lower in the control group, whereas it appeared similar in patients with PA and EH. Systolic and Diastolic blood pressure (BP), both measured in the clinic and during 24-hour ambulatory blood pressure monitoring (ABPM), were comparable in patients with PA and EH and were found to be statistically different from the patients in the control group. Mean known duration of hypertension was also similar between the PA and EH groups. No significant difference was found in the proportion of the different classes of anti-hypertensive drugs or in statin use. As expected, the plasma aldosterone concentration was significantly higher and plasma renin activity levels significantly lower in patients with PA as against patients with EH.

Pulse wave velocity (PWV), brachial and aortic augmentation index (Aix) data of PA and EH patients and of the control group are listed in Table 2. PA patients showed significantly higher values of PWV in comparison with the control group but were no different to those in EH patients. Mean Brachial AIx values were higher in the PA group and lower in the control group; however, no statistically significant difference was detected between each group. Similarly, there was no significant difference in the mean aortic AIx value between each group, the lower mean values being in the PA and in the control groups and the higher in the EH group. Aortic blood pressure values were found to be significantly different in the PA and EH groups when compared with normal subjects, whereas no difference was detected between the PA and the EH groups.

**Table 2:** Retinal microvascular and systemic macrovascular assessment in hypertension secondary to primary aldosteronism. Arterial stiffness parameters and retinal microvascular characteristics.

	Essential Hypertension (EH) (39) Mean ± SD	Primary Aldosteronism (PA) (30) Mean ± SD	Control group (35) Mean ± SD	F ratio (p value, ANOVA statistic)
Brachial Aix (%)	−21.9 ± 29.1 (EH vs PA: p:0.82) (EH vs control: p:0.81)	−23.4 ± 26,6 (PA vs control: 0.64)	−20.4 ± 25	0.0997 (p:0.905)
Aortic AIx (%)	26.9 ± 16.4 (EH vs PA: p:0.76) (EH vs control: p:0.73)	25.8 ± 13.5 (PA vs control: 1.00)	25.8 ± 11.7	0.0840 (p:0920)
PWV (m/s)	8.7 ± 2.1 (EH vs PA: p:0.30) (EH vs control: p:0.09)	9.2 ± 1.9 (PA vs control: 0.01)	7.7 ± 2.7	3.847 (p:0.024)
Retinal artery Diameter (µm)	103.6 ± 11.2 (EH vs PA: p:0.12) (EH vs control: p:0.47)	98.7 ± 14.2 (PA vs control: 0.06)	106.3 ± 16.7	2.376 (p:0.09)
Retinal Vein Diameter (µm)	134.2 ± 15.9 (EH vs PA: p:0.01) (EH vs control: p:0.96)	125.2 ± 14.6 (PA vs control: 0.04)	134.0 ± 19.1	3.031 (p: 0.049)
AVR (index)	0.84 ± 0.06 (EH vs PA: p:0.02) (EH vs control: p:0.19)	0.802 ± 0.07 (PA vs control: 0.001)	0.86 ± 0.07	6.555 (p:0.002)

Brachial AIx: Brachial Augmentation index; Aortic Aix: Aortic Augmentation index; PWV: Pulse Wave Velocity; AVR: arteriole-to-venule ratio. Mean ± S: Mean ± Standard Deviation.

The mean value of retinal artery diameter (RAD) was found to be lower in the PA group in comparison with the EH and control groups, although each comparison between the three groups did not reveal any significant difference. The lower retinal vein diameters (RVD) in the PA group compared with the EH and control groups was statistically significant. Similarly, a statistically significant reduction in the mean AVR value was found in the PA group in comparison with the EH group and normal subjects. The RVD and arteriole-to-venule ratios (AVR) did not differ between the EH and control groups.

The differences in the RAD, RVD and AVR were further analyzed in order to detect the possible influence of age, systolic blood pressure, estimated glomerular filtration rate and pulse pressure (Table 3). In particular, the differences in RVD between them PA group and the EH and control groups were lost after correction for each additional co-variable. In contrast, the PA group maintained the statistically significant difference in the AVR index when compared with the EH group and the control group.

**Table 3:** Retinal artery diameter, retinal vein diameter and AVR in Essential Hypertension and Primary Aldosteronism: analysis of variance and influence of the covariates: age, systolic blood pressure, eGFR and pulse pressure.

	ANOVA (F ratio, p value)	ANCOVA (F ratio, p value)			
		Age (years)	Systolic Blood Pressure (mmHg)	eGFR (ml/min)	Pulse Pressure (mmHg)
**Retinal artery diameter**	0.0997 (p:0.905)	1.61 (p:0.19)	2.85 (p:0.07)	1.58 (p:0.19)	2.87 (p: 0.08)
PA vs Control	p: 0.061	p:0.43	p:1.00	p:0.23	p:0.45
PA vs EH	p:0.126	p:0.10	p:0.77	p:0.47	p:0.70
EH vs control	p:0.476	p:1.00	p:1.00	p:1.00	p:1.00
**Retinal Vein diameter**		2.02 (p: 0.11)	2.02 (p: 0.11)	2.37 (p:0.07)	2.00 (p: 0.11)
PA vs Control	p: 0.04	p:0.12	p:0.17	p:1.00	p:0.13
PA vs EH	p: 0.017	p:0.08	p:0.08	p:0.72	p:0.08
EH vs control	p: 0.96	p:1.00	p:1.00	p:0.58	p:1.00
**AVR (index)**		4.72 (p:0.004)	4.33 (p:0.006)	5.32 (p:0.002)	4.34 (p:0.006)
PA vs Control	p: 0.001	p:0.001	p:0.006	p:0.0007	p: 0.003
PA vs EH	p: 0.021	p:0.061	p:0.056	p:0.015	p:0.06
EH vs control	p: 0.194	p:0.42	p:0.60	p:0.15	p:0.61

PA: primary aldosteronism. EH: essential hypertension. AVR: arteriole-to-venule ratio. eGFR: estimated glomerular filtration rate.

## Discussion

Retinal vascular imaging of the microcirculatory system has attracted considerable attention in the field of cardiovascular research. This is mainly due to the unique possibility of a direct and non-invasive observation of a micro-circulatory system which has high structural and functional homologies with other models, including the myocardial and cerebral systems^11–13^.

Recent studies have provided new insights into the role of some new parameters, including retinal vascular tortuosity, fractal dimension and bifurcation. Additional investigations also considered the AVR and its role as a risk factor for vascular complications secondary to essential hypertension^14–17^.

Notable vascular remodeling phenomena are also present in arterial hypertension secondary to primary aldosteronism^18–20^. This condition represents the most common endocrine form of secondary hypertension, amounting to 5–15%^21–23^ of all cases. It is of special interest that an increased level of aldosterone induces marked changes in the extracellular matrix due to collagen deposit, leading ultimately to an increase in arterial stiffness of the macrocirculation^4, 5^.

The aim of the current study was to evaluate the modifications of the microvascular retinal circulation in association with the alterations of macrocirculation in patients with arterial hypertension secondary to PA and EH, compared with a normotensive control group.

Our study pointed out that the average values of the AVR were statistically lower in subjects with PA than in patients with EH and in the control group, even after evaluation of age, systolic blood pressure, estimated glomerular filtration rate and pulse pressure as confounding factors. In addition, the AVR did not reveal a statistically significant difference between the group of patients with EH and the control group. At the same time, arterial stiffness analysis revealed a statistically significant increased value of PWV in the PA group, in comparison with the normotensive group, but not versus the group of patients with EH.

AVR consists of a ratio of the caliber of arterioles to venules. In the imaging analysis, it is not affected by magnification differences caused by camera lens adjustments and refractive errors^24^. A considerable drawback of the parameter is the loss of the contribution of the single variable. For example, a reduction in the AVR may be the consequence of a reduction in the arteriolar diameter or an increase in the retinal venular caliber or both. In our cohort of patients, the lowest arteriolar diameter values were found in the PA group, compared with the EH and normotensive groups, while the PA group also showed the lowest venule diameter values.

The narrowing of the retinal artery and widening of the retinal venular caliber are both involved in arterial hypertension. The mechanism linking the narrowing of the retinal artery and the development of hypertension is not well understood. However, several studies have demonstrated that increased peripheral vascular resistance is associated with an increased incidence of hypertension and a rise in blood pressure over time^25–29^. In addition, there is increasing evidence that retinal venular widening is strongly related to concurrent elevated BP levels, suggesting a dynamic component responsive to changes in the microcirculation^28, 30^. Thus, changes in the retinal microvasculature structure observed in the current study seem only partially to corroborate the clinical evidence that abnormalities in the vascular network may be more pronounced in patients with PA.

Our data show that PA patients had a greater retinal artery narrowing than EH and healthy subjects, although they did not achieve statistical significance. It is worth noting that a wider retinal venular caliber was not observed in the PA group in our investigation and that although a difference was measured in the RVD between the PA group and the EH and control groups, the difference was no longer significant after correction for the co-variables.

It should be borne in mind that our sample consisted of patients with recent onset of hypertension and that the changes in the retinal arterial and venular diameters may occur in a long-standing disease and might only appear as a time-related modification. In addition, smoking habits, obesity, statin use or the different classes of anti-hypertensive drugs are all variables able to influence the retinal vascular caliber. Although no difference was registered for all these parameters among the three groups, a different influence might be identified in a larger population study. Finally, we should consider the wide variability in the retinal venous caliber in patients with arterial hypertension, as demonstrated in the Rotterdam and MESA studies, where an increased venous diameter and a reduced venous diameter were found also to be associated with arterial hypertension^31, 32^.

Previous investigations provide evidence that some retinal microvascular abnormalities are strictly related to target end-organ damage. In particular, it has been reported that a wider retinal venular caliber is related to a risk of stroke rather than a narrower arteriolar caliber^33^ and that patients with PA show a higher incidence of stroke in comparison with matched EH patients^34^.

Only longitudinal studies, with a larger cohort of patients, a long-term follow-up and targeted histological studies, will be able to provide additional material to test the hypothesis linking the changing of the retinal venular caliber and the prevalence of stroke in patients affected by PA.

Analysis of arterial stiffness in the present study demonstrated that patients with PA had higher PWV values than patients with EH and normotensive patients, achieving a statistically significant difference only in comparison with the latter group, whereas no difference in the AIx values was found in each comparison. Although in previous studies patients with PA often displayed an increase in PWV and AIx in comparison with EH patients^18, 19, 35^, the absence of a meaningful difference in the PWV and the AIx parameters between PA patients and the HE group in our investigation is not so unexpected. Tsioufis *et al*. registered higher mean PWV values in patients with early diagnosis of hypertension secondary to PA than in HE patients^18^. However, the study failed to reach statistical significance and a shorter known-duration of hypertensive disease was suggested as the determining factor. As in our study, it is important to bear in mind the small sample used for the study and/or the early stage of the disease when interpreting the data.

The early artery remodeling noted in the present study’s PA group, compared with the control group, may be ascribed to the direct effect of high aldosterone on vascular smooth muscle cells, leading to functional impairment of arterial conduit function. Similarly, previous studies often failed to find differences in AIx values between PA, EH and the normotensive group^19, 36^. AIx is a complex index describing mainly wave reflections occurring at the branching of the arterioles and appears to be influenced by several confounding factors, including age, height, heart rate, gender, calcium levels, diastolic blood pressure and systolic blood pressure^37–44^.

Several variables may influence PWV, including aldosterone plasma level, weight, age, use of anti-hypertensive drugs or statins^45–50^. Our population study consisted of patients with PA and EH who were well matched for all these baseline parameters. The reason for the absence of a relevant difference in PWV between PA and EH groups remains to be determined. We hypothesize that the sample size and disease duration could themselves have influenced our results.

Taken together, our data suggest that retinal microvascular changes and macrovascular modifications secondary to PA appear to manifest differently in comparison with EH. In particular, AVR reduction appeared earlier in PA than in EH and in the normotensive group, while PWV was not yet found to be significantly modified. A peripheral morphological change might be a more reliable indicator of vascular remodeling than the assessment of macrovascular change measured by means of PWV and AIx parameters.

AVR is currently under investigation owing to its association with an increased risk of target organ damage, especially left ventricular hypertrophy^51, 52^. It is worth noting that AVR appears to be an independent risk factor for stroke. AVR may have a further useful application in relation to the observation that the AVR parameter may increase in response to antihypertensive treatment by modifying the retinal arteriole diameter, expressing an improvement in retinal microcirculation^53^. In this context, AVR may be a useful indicator as a therapeutic target capable of reflecting the effectiveness of antihypertensive drug treatment in controlling high BP values, PA included.

Moreover, data acquired in the current study will serve as the basis for a future study exploring the dynamic proprieties of the retinal microcirculation by means of a dynamic retinal vessel analyzer. There is growing evidence suggesting that, independently of its effects on blood pressure, aldosterone impairs vascular function through the suppression of nitric oxide formation and that this impairment in endothelium-dependent vascular reactivity is partially reversible through chronic mineralocorticoid receptor blockade. The dynamic retinal vessel analyser offers an accurate and non-invasive method of measuring vessel motility, assessed in terms of retinal vessel response to flickering light stimulation, and can be performed on the main vessels of the posterior pole. Flickering light stimulation of the retina is an original technique that has been used in healthy subjects to investigate the process of neurovascular coupling. Such a mechanism enables the retina to regulate blood flow in response to neural activity. The increase in neural activity induced by flicker stimulation leads to retinal arterial and venous dilation by releasing vaso-active factors, such as nitric oxide, from neural and endothelial cells. In the light of these considerations, the reactivity of the retinal microcirculation may be taken as a model to evaluate the efficacy of specific anti-hypertensive drugs^54–56^.

One of the present study’s strengths lies in its being based on homogeneous groups whose demographic and clinical characteristics appear well matched.

However, as mentioned above, the sample size examined in the current study could be considered a limiting factor. Indeed, bearing in mind that retinal vessel caliber varies widely and that hypertension secondary to PA amounts to about 10% of the secondary forms of arterial hypertension, it is essential to perform a study with adequate power and a larger cohort of patients to confirm the outcomes of our pilot study.

An advantage of computer assisted analysis of retinal microvascular modification is the high inter-observer and intra-observer reliability provided by a trained rater^57^. Despite significant improvement in imaging technology, there are still confounding factors in imaging acquisition and interpretation, including refractive error and axial length, which may be partially compensated by AVR assessment.

One additional limitation of this cross-sectional study is the relatively short known-duration of the patients’ hypertension. The changes in arterial stiffness and retinal microcirculation described in this analysis may be distinctive features of a population of subjects with the same characteristics, although these changes evolve at different stages of the disease.

Finally, we considered the assessment of a single point of the retinal temporal artery and of the retinal temporal vein as representative of the modifications in the retinal circulation. More generalized indexes, such as the central retinal arterial equivalent and venular equivalent, might be more suitable indicators of peripheral microvascular abnormalities.

To our knowledge, this is the first investigation describing the assessment of the retinal microvasculature system in combination with the macrovascular compartment in patients affected by PA. Our data demonstrate that the AVR is greatly modified in PA in comparison with EH and could represent an early and more reliable indicator of vascular remodeling. Although arterial stiffness alteration is more usually associated with PA than with EH, in our study it appeared only slightly affected, displaying an unremarkable increase in the mean PWV value.

Longitudinal studies with a large population are required to confirm our preliminary data and to verify the behavior of AVR in response to treatment, including adrenalectomy or medical treatment.

## Methods

We performed a case-control observational study on consecutive adult patients affected by systemic arterial hypertension referred to the Specialized Center of Secondary Hypertension of the Department of Internal Medicine and Medical Specialities (“La Sapienza“ University, Rome, Italy) since December 2012. Each consecutive patient with PA and EH and no intercurrent state of illness was offered the chance to be included in the current observational study, highlighting the investigative nature of the study and the absence of any risk in the data acquisition procedures.

The study protocol adhered to the tenets of the Declaration of Helsinki and was approved by the Institutional Review Board of the G.B. Bietti Foundation and each patient gave signed informed consent before enrolment.

Inclusion criteria were defined as follows: (a) diagnosis of PA, based on criteria of the Endocrine Society Clinical Practice Guideline^58^ and including raised biochemical markers of hyperaldosteronism; (b) EH diagnosed by clinic and ambulatory blood pressure monitoring (ABPM) in accordance with the criteria of the guidelines of the European Society of Hypertension (ESH) and the European Society of Cardiology (ESC)^59^; (c) the control group was represented by a population of healthy subjects matched for age and gender, not taking any medicines and with no significant medical history (matching was done by groups of subjects). Normal subjects were recruited on a voluntary basis and were mainly hospital workers or were part of the administrative or medical staff.

Major exclusion criteria were defined as follows: secondary prevention for cardiovascular diseases, heart failure, liver cirrhosis, diabetes, kidney failure (Glomerular Filtration Rate (GFR) MDRD < 60 ml/min), cardiac arrhythmias, severe obesity (Body Mass Index (BMI) > 35 kg/m2), peripheral artery disease (PAD). Additional exclusion criteria comprised a history of any ocular condition able to confound an appropriate fundus ocular assessment and especially a history of glaucoma, presence of cataract or media opacities preventing a proper assessment of the retina and the acquisition of the image of the fundus, pathologic myopia and any other type of retinal disorder.

Arterial stiffness was assessed by examining the AIx and the PWV. Measurements were carried out by using a TensioClinic arteriograph system (TensioMed Kft., Debrecen, Hungary). Details of this device have been previously described^60^. This technique is based on the fact that the contraction of the myocardium generates pulse waves in the aorta. The pulse wave goes to the arm (first wave), where the cuff is located, then to the aorta. Part of the wave is reflected at the bifurcation of the aorta, goes back, adds to the first and is sensed by the cuff. The first wave is reflected at the bifurcation of the aortic wall; a second, reflected wave therefore appears as a late systolic peak. The morphology of this second wave depends on the stiffness of the large artery, the reflection time at 35 mmHg suprasystolic pressure of the brachial artery (RT S 35) and the peripheral resistance-dependent amplitude.

The cuff collects a pulse wave that contains both the first and reflected waves. The software calculates PWV and AIx from this combined wave. AIx is calculated from the amplitudes of the first and second wave and represents the pressure difference between the pressure of the late systolic peak and that of the early systolic peak divided by the pulse pressure. PWV is the quotient of the jugular fossa–symphysis distance and RT S35 in meters per second. The jugular fossa–symphysis distance is anatomically identical to the distance between the aortic trunk and the bifurcation. All the measurements were performed by a single fully-trained operator in a quiet room and after the participant had rested 10 minutes in a supine position.

Each patient received a comprehensive ophthalmological assessment, involving a visual acuity and slit-lamp examination, Goldmann applanation tonometry and ocular fundus evaluation. All patients were imaged by a Retinal Vessel Analyzer (RVA) by positioning the subject in front of a non-midriatic camera (Topcon TRC-NV2000) to obtain a retinographic image (45°). The images were then processed by IMEDOS software measuring the caliber of vessels emerging from the optic disc. The RVA produces continuous and automated analysis of the vessels combined with correction for small eye movements. Using an adaptive algorithm to calculate the size of the vessels (VesselMap 2-Z), this system combines high reproducibility with high temporal and spatial resolution.

**Figure 1:**
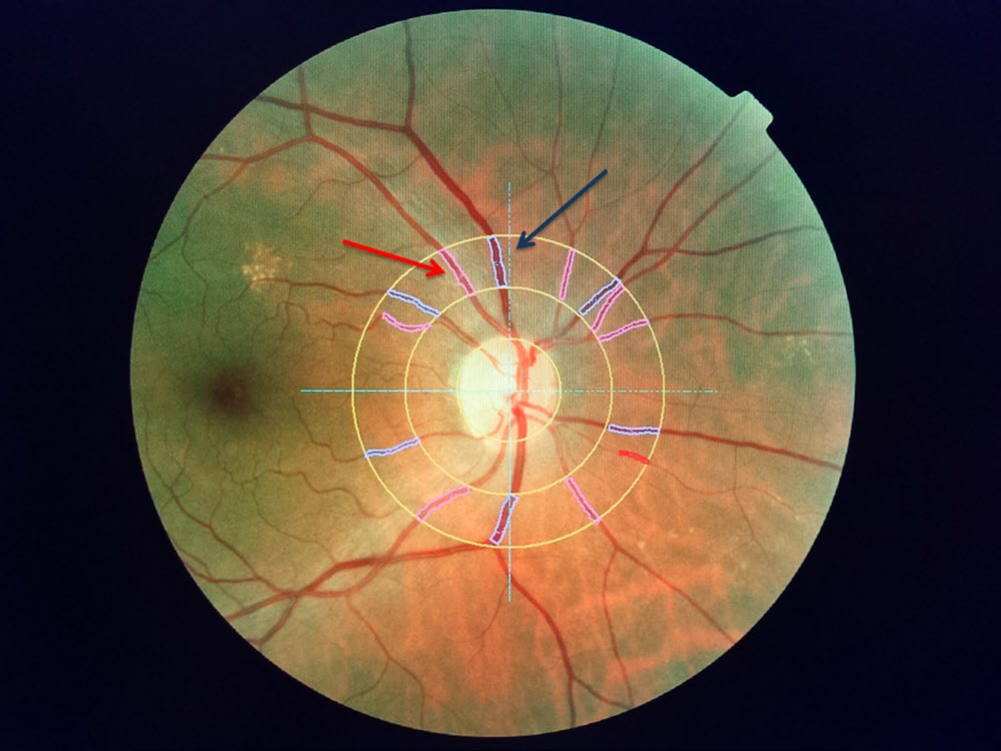
Example of an image obtained by retinal vessel analyzer (RVA) and then processed by Imedos software that provided the caliber of the vessels emerging from the optic disc. The assessments of the arterial calibre and of the vein calibre were taken between 1 and 2 disc diameters from the margin of the optic disc, in correspondence with the major superior temporal arteries (Red Arrow) and vein (Blue Arrow) of the right eye.

Measurements of RAD, RVD and AVR were taken between 1 and 2 disc diameters from the margin of the optic disc. The major superior temporal arteries and vein of the right eye were studied (Fig. 1). In view of the high reproducibility of the procedure calculated in a preliminary exploratory analysis performed in patients with EH and normal subjects (coefficient of variation below 4% for day-to-day variability) and on the basis of data in the literature, only one acquisition session was scheduled for each subject. One single measurement was performed by positioning the caliber at the outer margin of the first circle delimitating the optic disc^61, 62^. The choice was based on the results of a previous study demonstrating the high correlation in vascular morphology between the left and the right eye^63^.

In addition to AIx, PWV, RAD, RVD and AVR, further data collection for the study included demographic characteristics (age, gender, known duration of hypertension, smoking habits, weight, height, BMI calculation, aortic blood pressure) and laboratory testing (glycemia, serum potassium level, creatinine, plasma aldosterone, and the plasma aldosterone/plasma renin activity ratio (PA/PRA)).

The Shapiro Wilk Test was used to check the assumption of normality of the variables. The Anova statistical test was used for the comparison of continuous variables in the three groups applying the Student-Newman-Keuls correction, while the χ² test was used for categorical variables. The adjustments for the co-variables (age, systolic blood pressure, estimated glomerular filtration rate and pulse pressure) were performed applying the Ancova statistical test with Bonferroni correction; each variable was separately analyzed and not included in a model and each comparison was individually adjusted for each co-variable. All statistical comparisons were performed using 2-tailed significance tests, with p < 0.05 considered statistically significant. MedCalc Statistical Software (version 17.1, Ostend, Belgium) was used for all analyses.

## References

[1] Rossi GP (2006). PAPY Study Investigators. A prospective study of the prevalence of primary aldosteronism in 1,125 hypertensive patients. J Am Coll Cardiol..

[2] Milliez P (2005). Evidence for an increased rate of cardiovascular events in patients with primary aldosteronism. J Am Coll Cardiol..

[3] Strauch B (2008). Adrenalectomy improves arterial stiffness in primary aldosteronism. Am J Hypertens..

[4] Rizzoni D (2006). Changes in extracellular matrix in subcutaneous small resistance arteries of patients with primary aldosteronism. J Clin Endocrinol Metab.

[5] Holaj R (2015). Long-term effect of specific treatment of primary aldosteronism on carotid intima-media thickness. J Hypertens..

[6] Laurent S (2001). Aortic stiffness is an independent predictor of all-cause and cardiovascular mortality in hypertensive patients. Hypertension..

[7] Laurent S (2003). Aortic stiffness in an independent predictor of fatal stroke in essential hypertension. Stroke..

[8] Rosa J (2012). Peripheral arterial stiffness in primary aldosteronis. Physiol Res..

[9] Catena C (2008). Cardiovascula outcomes in patients with primary aldosteronism after treatment. Arch Intern Med..

[10] Rizzoni D (2012). Relationship between media-to-lumen ratio of subcutaneous small arteries and wall-to-lumen ratio of retinal arterioles evaluated noninvasively by scanning laser Doppler flowmetry. J Hypertens..

[11] Tso MO, Jampol LM (1982). Pathophysiology of hypertensive retinopathy. Ophthalmology..

[12] Goto I, Katsuki S, Ikui H, Kimoto K, Mimatsu T (1975). Pathological studies on the intracerebral and retinal arteries in cerebrovascular and noncerebrovascular diseases. Stroke..

[13] Tanaka M (1987). Quantitative analysis of narrowings of intramyocardial small arteries in normal hearts, hypertensive hearts, and hearts with hypertrophic cardiomyopathy. Circulation..

[14] Witt N (2006). Abnormalities of retinal microvascular structure and risk of mortality from ischemic heart disease and stroke. Hypertension..

[15] Patton N (2006). Retinal image analysis: concepts, applications and potential. Prog Retin Eye Res..

[16] Gould DB (2006). Role of COL4A1 in small-vessel disease and hemorrhagic stroke. N Engl J Med..

[17] Mainster MA (1990). The fractal properties of retinal vessels: embryological and clinical implications. Eye..

[18] Tsioufis C (2008). Myocardial and aortic stiffening in the early course of primary aldosteronism. Clin Cardiol..

[19] Strauch B (2006). Increased arterial wall stiffness in primary aldosteronism in comparison with essential hypertension. Am J Hypertens..

[20] Rossi GP (2008). Vascular remodeling and duration of hypertension predict outcome of adrenalectomy in primary aldosteronism patients. Hypertension.

[21] Takeda Y, Karashima S, Yoneda T (2011). Primary aldosteronism, diagnosis and treatment in Japan. Rev Endocr Metab Disord..

[22] Rossi GP (1996). Changes in left ventricular anatomy and function in hypertension and primary aldosteronism. Hypertension..

[23] Rossi GP (2010). Prevalence and diagnosis of primary aldosteronism. Curr Hypertens Rep..

[24] Cheung CY, Ikram MK, Sabanayagam C, Wong TY (2012). Retinal microvasculature as a model to study the manifestations of hypertension. Hypertension..

[25] Levy BI, Ambrosio G, Pries AR, Struijker-Boudier HA (2001). Microcirculation in hypertension: a new target for treatment?. Circulation..

[26] Wong TY (2001). Retinal microvascular abnormalities and their relationship with hypertension, cardiovascular disease, and mortality. Surv Ophthalmol..

[27] Mulvany MJ (1991). Are vascular abnormalities a primary cause or secondary consequence of hypertension?. Hypertension..

[28] Liew G, Wang JJ, Mitchell P, Wong TY (2008). Retinal vascular imaging: a new tool in microvascular disease research. Circ Cardiovasc Imaging..

[29] Tanabe Y (2010). Retinal arteriolar narrowing predicts 5-year risk of hypertension in Japanese people: the Funagata study. Microcirculation..

[30] Patton N, Aslam T, Macgillivray T, Dhillon B, Constable I (2006). Asymmetry of retinal arteriolar branch widths at junctions affects ability of formulae to predict trunk arteriolar widths. Invest Ophthalmol Vis Sci..

[31] Wong TY (2006). Retinal vascular caliber, cardiovascular risk factors, and inflammation: the multi-ethnic study of atherosclerosis (MESA). Invest Ophthalmol Vis Sci..

[32] Ikram MK (2004). Are retinal arteriolar or venular diameters associated with markers for cardiovascular disorders? The Rotterdam Study. Invest Ophthalmol Vis Sci..

[33] McGeechan K (2009). Prediction of incident stroke events based on retinal vessel caliber: a systematic review and individual-participant meta-analysis. Am J Epidemiol.

[34] Mulatero P (2013). Long-term cardio- and cerebrovascular events in patients with primary aldosteronism. J Clin Endocrinol Metab..

[35] Bernini G (2008). Arterial stiffness, intima-media thickness and carotid artery fibrosis in patients with primary aldosteronism. J Hypertens..

[36] Mark PB (2014). Alterations in vascular function in primary aldosteronism: a cardiovascular magnetic resonance imaging study. J Hum Hypertens..

[37] Catalano M (2014). Aortic augmentation index in patients with peripheral arterial disease. J Clin Hypertens (Greenwich)..

[38] Pierce GL (2013). Arterial stiffness and pulse-pressure amplification in overweight/obese African-American adolescents: relation with higher systolic and pulse pressure. Am J Hypertens..

[39] Budimir D (2012). Sex-specific association of anthropometric measures of body composition with arterial stiffness in a healthy population. Med Sci Monit..

[40] Janner JH, Godtfredsen NS, Ladelund S, Vestbo J, Prescott E (2012). The association between aortic augmentation index and cardiovascular risk factors in a large unselected population. J Hum Hypertens..

[41] Chirinos JA (2011). Ethnic differences in arterial wave reflections and normative equations for augmentation index. Hypertension..

[42] Lieber A (2010). Aortic wave reflection in women and men. Am J Physiol Heart Circ Physiol..

[43] Kim OY, Baek SH, Lee YJ, Lee KH (2010). Association of increased hair calcium levels and enhanced augmentation index (AIx): a marker of arterial stiffness. Biol Trace Elem Res..

[44] Ayer JG, Harmer JA, Marks GB, Avolio A, Celermajer DS (2010). Central arterial pulse wave augmentation is greater in girls than boys, independent of height. J Hypertens..

[45] Kwon BJ (2013). Comparison of the efficacy between hydrochlorothiazide and chlorthalidone on central aortic pressure when added on to candesartan in treatment-naïve patients of hypertension. Hypertens Res..

[46] Kanaki AI (2013). Effects of low-dose atorvastatin on arterial stiffness and central aortic pressure augmentation in patients with hypertension and hypercholesterolemia. Am J Hypertens.

[47] Guo JQ, Wang HY, Sun NL (2013). Effect of aliskiren on arterial stiffness, compared with ramipril in patients with mild to moderate essential hypertension. Chin Med J (Engl)..

[48] Leopold JA (2013). Cellular and molecular mechanisms of arterial stiffness associated with obesity. Hypertension..

[49] Kudo U (2013). Influence of obesity on blood pressure and arterial stiffness in the early teens. Obes Res Clin Pract..

[50] Li S (2014). Sex and Race (Black-White) Differences in the Relationship of Childhood Risk Factors to Adulthood Arterial Stiffness: The Bogalusa Heart Study. Am J Med Sci..

[51] Coll-de-Tuero G (2014). Retinal arteriole-to-venule ratio changes and target organ disease evolution in newly diagnosed hypertensive patients at 1-year follow-up. J Am Soc Hypertens..

[52] Meazza R (2014). Target organ damage in hypertensive patients: correlation between retinal arteriovenular ratio and left ventricular geometric patterns. J Hum Hypertens..

[53] Antonio PR (2014). Factors associated with changes in retinal microcirculation after antihypertensive treatment. J Hum Hypertens..

[54] Falsini B, Riva CE, Logean E (2002). Flicker-evoked changes in human optic nerve blood flow: relationship with retinal neural activity. Invest Ophthalmol Vis Sci.

[55] Riva CE, Logean E, Falsini B (2005). Visually evoked hemodynamical response and assessment of neurovascular coupling in the optic nerve and retina. Prog Retin Eye Res..

[56] Nishizaka MK, Zaman MA, Green SA, Renfroe KY, Calhoun DA (2004). Impaired endothelium-dependent flow-mediated vasodilation in hypertensive subjects with hyperaldosteronism. Circulation..

[57] Wang JJ (2008). The long-term relation among retinal arteriolar narrowing, blood pressure, and incident severe hypertension. Am J Epidemiol..

[58] Funder JW (2008). Endocrine Society. Case detection, diagnosis, and treatment of patients with primary aldosteronism: an endocrine society clinical practice guideline. J Clin Endocrinol Metab.

[59] Mancia G (2007). The task force for the management of arterial hypertension of the European Society of Hypertension, The task force for the management of arterial hypertension of the European Society of Cardiology. 2007 Guidelines for the management of arterial hypertension: The Task Force for the management of arterial hypertension of European Society of Hypertension (ESH) and the European Society of Cardiology (ESC). Eur Heart J.

[60] Hung CS (2009). Using brachial-ankle pulse wave velocity to associate arterial stiffness with cardiovascular risks. Nutr Metab Cardiovasc Dis.

[61] Hubbard LD (1999). Methods for evaluation of retinal microvascular abnormalities associated with hypertension/sclerosis in the atherosclerosis risk in communities study. Ophthalmology..

[62] Sacu S (2011). Response of retinal vessels and retrobulbar hemodynamics to intravitreal anti-VEGF treatment in eyes with branch retinal vein occlusion. Invest Ophthalmol Vis Sci..

[63] Leung H (2003). Computer-assisted retinal vessel measurement in an older population: correlation between right and left eyes. Clin Experiment Ophthalmol..

